# Effects of Massage Therapy on the Development of Babies Born with Down Syndrome

**DOI:** 10.1155/2020/4912625

**Published:** 2020-05-06

**Authors:** Elena Pinero-Pinto, María-Luisa Benítez-Lugo, Raquel Chillón-Martínez, Manuel Rebollo-Salas, Lorena-María Bellido-Fernández, José-Jesús Jiménez-Rejano

**Affiliations:** Department of Physiotherapy, University of Seville, 41009 Seville, Spain

## Abstract

**Objective:**

To determine the short-term effects of infant massage on the development of Down syndrome babies.

**Materials and Methods:**

The study compared two groups (intervention and control), each with 16 babies with Down syndrome between 4 and 8 months old. The variables developmental age and developmental quotient were measured at two distinct time points, at pretest and after 5 weeks, using the Brunet-Lézine Early Childhood Psychomotor Development revised scale. This scale measures the variables of age and development quotient in a partial way (motor, visual-motor coordination, language, and social development) and in a global way. The experimental group received infant massage, applied by the parents, during these 5 weeks, every day for at least 10 minutes. The massage protocol was based on the methodology created by Vimala McClure. The control group received it after 5 weeks.

**Results:**

All developmental variables were improved in the experimental group but not in the control group. There were significant differences in developmental age between the two groups, and this outcome was better in the experimental group (*p* < 0.001). The 2-by-2 mixed-model analysis of variance indicates a statistically significant group-by-time interaction for all development quotients, both partial and global (*p* < 0.001), which was significantly higher in the experimental group than in the control group.

**Conclusion:**

Infant massage therapy improves the development of babies with Down syndrome in the short term.

## 1. Introduction

Down syndrome is the most common developmental disorder involving intellectual disability [1]. The development of children with Down syndrome is affected by the presence of extra information in chromosome 21, and abnormal gene expression leads to changes in brain function [1–3].

There are global alterations in the development process, which affect behavioral, motor, language, cognitive, autonomy, and social phenotypes. The alterations found in different areas of development are not always proportional, since each area influences and is influenced each other [4]. The cognitive development of individuals with Down syndrome is characterized by limited mental ability and difficulty in processing information [5]. Intellectual deficits cause alterations in attention, memory, language acquisition, and other mental abilities. The limitations in the exploration of the environment also directly affect the development of language and sociability [4]. Children with Down syndrome also have a delay in motor development, and their movement patterns may be affected. Generalized hypotonia and ligamentous hyperlaxity strongly affect motor development because these conditions impair movements and the maintenance of postures [6, 7].

There are many studies on infant massage applied to premature babies, which indicate that it reduces the length of hospital stay and improves sleep, among other things [8, 9].

In children with Down syndrome, Hernandez-Reif et al. [10] described the increase in fine and gross motor function and less severe hypotonicity, after massage therapy. This study was carried out with a sample of 21 children with Down syndrome (mean age: 2 years), applying infant massage to the experimental group and reading stories to the control group. The frequency of application of the massage was 0.5 hours twice a week for two months. Purpura et al. [1] also reported that infant massage appears to positively affect the maturation of visual functions in babies with Down syndrome. Their sample consisted of 20 babies between 2 and 4 months of age, applying the Vimala McClure (International Association of Infant Massage) technique. The intensity of the application was 15 minutes daily made by the parents for 2 months. Lima [11] evaluated infant massage in children with Down syndrome as a technique to improve motor development, obtaining significant results with Shantala technique. In this case the sample is small, applying the technique in only 5 mother-child dyads. Silva et al. [12] also investigated the effects of infant massage on the development of motor skills in children, including some with Down syndrome. Qigong technique is based on Chinese Medicine to clear the channels. The results were also positive with this technique, which was carried out on a sample of 14 children with Down syndrome aged less than 4 years. This technique is also applied by parents who previously learned it by training for 5 months. Time is also spent explaining adaptations and possible reactions, among other things, to parents.

For all this, infant massage could be part of the preventive care of physiotherapy, as a complement to early care programs, ensuring the general development of the child, such as gross motor function [13, 14].

The objective of this study was to determine the effects of implementing infant massage therapy on the global development of babies with Down syndrome, in order to compare the effect in different areas of development. Previous studies only looked at a specific area of development. Researchers think that it is important to limit the age range to the first months of development, with a significant sample, where the evolution is faster.

## 2. Materials and Methods

### 2.1. Study Design

An analytical, longitudinal, prospective, experimental, and multicenter study is proposed. This randomized controlled clinical trial evaluated the effect of infant massage therapy applied by parents on the development of babies with Down syndrome. This study was registered in Clinical Trials under protocol no. NCT03084497.

### 2.2. Sample Selection and Scope of the Study

The sample consisted of babies with Down syndrome attending different institutions and early childhood intervention centers. These centers are dedicated to therapy to promote the development of people with developmental disorders through psychology, physiotherapy, and speech therapy.

### 2.3. Selection Criteria

#### 2.3.1. Inclusion Criteria

Babies with Down syndrome aged 4 to 8 months who received early childhood intervention were included. Most motor development occurs in the first year of life. Assessing a baby before 4 months is not reliable from a developmental point of view. Performing infant massage in children who have acquired the sitting is quite complex, because they want to explore and move. Therefore, the research age has been limited to the range between 4 and 8 months.

#### 2.3.2. Exclusion Criteria

Babies with Down syndrome with untreated pathologies of the heart or kidneys or digestive diseases, babies with Down syndrome with another developmental disorder, adopted or fostered children with Down syndrome, premature babies with Down syndrome, and families that had previously received the infant massage therapy course were excluded.

**Figure 1:**
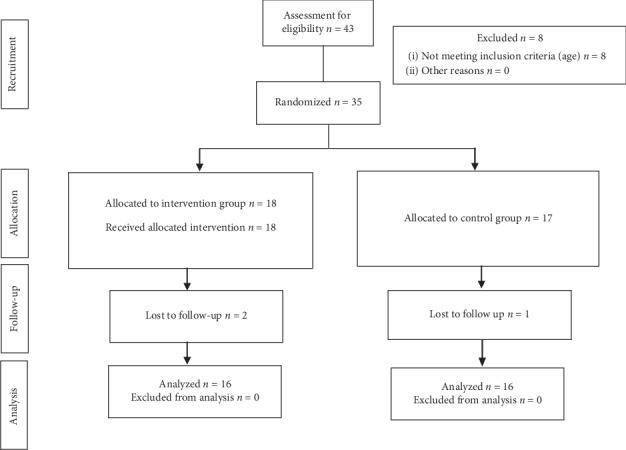
Flowchart of progress through the phases of the clinical trial of two groups, following CONSORT 2010 Declaration.

A consecutive nonprobabilistic sampling was conducted by contacting 43 families, of which eight did not participate because the babies had exceeded the age of 8 months at the time of the first evaluation. Three other families were excluded once the study began: two babies were hospitalized, so the second evaluation could not be performed 5 weeks after the first one; in the other case, the family did not attend all the sessions of the course. Of these three families, two belonged to the experimental group and one to the control group (Figure 1).

Therefore, the sample size was composed of 32 babies with Down syndrome. The free software G∗power version 3.1 was used to calculate the required sample size. The data provided were *α* error of 0.05 (confidence level [CI] of 95%), *ß* error of 0.2 (power of the study of 80%), large Cohen effect size (1.56) using a pilot study [14], sample size ratio of the two groups (N2/N1) equal to 1, and two-tailed hypothesis. Under these conditions, the estimated sample size was 28 babies (14 in each group). The effect size was reduced to 1.05 because a total of 32 subjects (16 in each group) were included. This work has a sample size in consonance with other similar studies such as the population reported by Purpura et al. [1].

Participants were assigned to each group (intervention or control) by random sampling stratified by gender. Concealed allocation was performed by method of sequentially numbered sealed opaque envelopes. Data entry person (assessor) remained blind to treatment allocation.

The mean age of the 32 babies was 155.72 ± 39.46 days, with a minimum of 117 days and maximum of 235 days. Of the 32 babies, 21 (65.6%) were boys and 11 (34.4%) were girls.

### 2.4. Study Variables

#### 2.4.1. Independent Variable

Infant massage therapy vs. no infant massage in babies with Down syndrome.

#### 2.4.2. Dependent Variables

The following were discrete quantitative variables measured using the Brunet-Lézine Early Childhood Psychomotor Development revised scale: (1) global developmental age in days, (2) motor developmental age in days, (3) visual-motor coordination age in days, (4) language developmental age in days, (5) social developmental age in days. The following were continuous quantitative variables measured as a function of the developmental age in days using the Brunet-Lézine Early Childhood Psychomotor Development revised scale: (1) global development quotient, (2) motor development quotient, (3) visual-motor development quotient, (4) language development quotient, and (5) social development quotient.

### 2.5. Measuring Instruments

Brunet-Lézine Early Childhood Psychomotor Development revised scale [15] was used. The scale aims to assess children aged between 2 and 30 months as to the following areas of development: motor development, visual-motor coordination, language, and sociability. Four separate developmental quotient subscores can be calculated for children aged 2 to 30 months. A global developmental quotient score results from the combination of the Brunet-Lézine scale subscores, with a mean norm of 100.

The choice of items is based on the chronological age of the child. Depending on the age of the child, the series of tests (10 items) of the corresponding level are applied, returning to lower levels in case of failure (even in a single item) and continuing otherwise, until there is a complete failure in an age level (10 items).

Before starting the correction, it is necessary to mention that the scale protocol serves to report the child's results in terms of approval (+) or disapproval (−). It also serves to calculate the score in points, the ages of development, and the development quotients and design the profile.

Once the scale is administered, the points are obtained by adding the items obtained in each subscale. Total points are obtained by adding the points of the four subscales. To calculate the development ages, the point conversion table to partial and global development ages is used. To get the development quotient, the following formula is used: (development age/chronological age) × 100.

Regarding the values of sensitivity to change in the study carried out by the authors of the scale [15], large effect sizes were obtained for all the subscales and for the global scale. Measurements on successive age pairs between 4 and 8 months were compared. Cohen's d for the motor development subscale is between 1.11 and 2.55. In the case of the visual-motor coordination subscale, Cohen's d is between 1.73 and 2.55. For the language subscale, the values are between 1.26 and 1.78, and for the social subscale, between 1.20 and 1.93. For global development, values range from 1.94 to 2.96. These data indicate that the scale is sensitive to change in scores.

The authors also determined the test-retest reliability through the stability coefficient. They found values of 0.89 for the motor QD, 0.74 for the visual-motor coordination QD, 0.87 for the language QD, 0.73 for the social QD, and 0.89 for the global QD. The mean value is 0.82 measured in a population of 79 subjects. The results show great stability over time, since a coefficient of 0.70 is considered satisfactory and that higher than 0.80 indicates good stability.

Internal consistency was calculated, determining the value of the Cronbach alpha coefficient. A high correlation was obtained from 4 months of age, ranging between 0.69 and 0.87. These data indicate that the test is reliable from 4 months of age.

### 2.6. Study Procedure

The families that met the selection criteria were contacted via early care centers and the Associations of Parents of Children with Down Syndrome of Spain. These families were contacted by e-mail and phone calls to assess whether their babies had the desired features. Data on the study were sent to the care centers to inform the families. The families interested in participating in the study were contacted by telephone to provide further information and clarify doubts. The families that accepted the terms of the study were asked to sign informed consent. After that, the families were randomly assigned to the intervention or control group using the sealed envelope technique. At the time of inclusion in each group, the following data were collected and transferred to a record sheet prepared for this purpose: personal data from the child and parents, personal history of the child, early childhood intervention center to which the infant was assigned, study group to which the child was assigned, and tables with the scores of the evaluations. Both experimental and control groups were evaluated in two moments with the same scale with 5 weeks of difference between the measurements. During those five weeks, the experimental group received a weekly infant massage class led by a physical therapist and applied daily by their parents at home. The control group did not receive this intervention. Both groups continued to attend their weekly early childhood intervention sessions (one session per week of physiotherapy in early family-centered care). A researcher who was external to the intervention with infant massage administered the Brunet-Lézine scale in an attempt to provide a more objective point of view regarding the impact of relational challenges on the babies.

If we consider that massage is beneficial for the infant, for ethical reasons, the families from control group were recommended to participate in the course after investigation.

### 2.7. Content and Administration of Infant Massage Intervention

After the initial assessment, babies from experimental group participated in a 5-week infant massage therapy course with their parents, as described below.

The course, led by a physical therapist, consists of five sessions (one per week) of 90 minutes of time, with theoretical classes and practical sessions of infant massage. Parents practiced it daily at home. The first 15 minutes of each session are used to ask about the practice of parents at home, as well as to resolve possible doubts of previous weeks. Subsequently, the corresponding technique is taught to all families. The therapist must confirm that everyone performs it correctly. Time is left to practice. This corresponds to 45 minutes for session. Finally, the theory of infant massage (benefits, baby reflexes that appear during massage, contraindications, and adaptations for older children through play) is explained. New doubts are also resolved. This is about 30 minutes of the session.

The massage protocol was based on the methodology and program of the International Association of Infant Massage (IAIM), created by Vimala McClure [13]. The technique resulted in an overall massage time of approximately 10–15 minutes. The massage protocol was adapted by a physical therapist who was certified by IAIM. First pressed sweet almond oil was used to assist with ease of skin-to-skin contact during moderate pressure massage. During massage, each infant received 2 repetitions of each movement. Massage occurred over 1-minute intervals with application of 10 strokes, lasting approximately 3 seconds each, for each of the body areas receiving massage. The massage took place in a floor pad using the following sequence: with baby in supine, the baby was stroked (1) from the foot to the thigh on both legs, (2) on abdomen clockwise, (3) on chest, (4) from shoulder to the hand, (5) on face from forehead to chin, and, with the baby in prone, (6) from the head to the end of the back. Parents received one session a week of training in infant massage for 5 weeks. Each week, parents performed daily the massage learned at home for 10 minutes. All parents who completed the full course claimed to have practiced it daily. Each session they were asked to explain what they had practiced since the previous week. In addition, they received a booklet with the description and images of the movements learned after each session to practice at home.

After completing the massage therapy course, the babies were subjected to a final evaluation.

### 2.8. Data Analysis

The data were organized and analyzed using the SPSS version 24.0 for Windows (SPSS, Chicago, United States). The effectiveness of the applied intervention was compared between the two groups. For this purpose, the evaluations were made at the beginning (pretest) and at the end of the intervention (after 5 weeks of therapy (posttest)). The Shapiro–Wilk test was used to assess the normality of the variables. Dependent and sociodemographic variables were analyzed descriptively. Qualitative variables were expressed as counts and proportions, whereas quantitative variables were expressed as means and standard deviations or medians and interquartile ranges. The homogeneity of the dependent variables at pretest and sample distribution by gender, age, presence of siblings, and parents who actively participated in the therapy and who answered the interview was also analyzed. Student's *t*-test for independent samples or Welch *t*-test was used for quantitative variables with normal distribution, and the Mann–Whitney *U* test was used for quantitative variables with nonnormal distribution. The Pearson chi-square test or Fisher exact test was used for qualitative variables. The Wilcoxon signed rank test and Student's *t*-test for related samples were used to assess differences between pretest and posttest in each group. The differences in the variables related to partial and global developmental age between pretest and posttest (“variable differences”) in the two groups were calculated using the Mann–Whitney *U* test. Finally, separate 2-by-2 mixed-model analyses of variance were used to examine the effects of treatment on development quotients as dependent variables, with group (intervention or control) as the between-subjects variable and time (pretest and posttest) as the within-subjects variable. The hypothesis of interest was the group-by-time interaction at an a priori alpha level of 0.05. The effect size of the observed differences was estimated by calculating the partial eta squared (*η*^2^). The effects of the applied intervention were evaluated on a per-protocol basis. All statistical tests were performed using a 95% CI (*p* < 0.05).

## 3. Results

There were no significant intergroup differences in the variables analyzed before the interventions; i.e., the two groups were homogeneous at pretest (Table 1).

The effects of infant massage for experimental group and the changes between two measurements for control group on developmental age are shown in Table 2. In the control group, there were significant differences between pretest and posttest for the global developmental age, motor developmental age, visual-motor coordination age, and language developmental age. However, there were no significant differences in social developmental age in this group. In the experimental group, there were significant differences in all studied variables. Developmental age was higher in the experimental group in all cases (*p* < 0.001).

There were no significant differences in the variables (partial and global) for the development quotients in the control group (see Table 3). In contrast, there were significant differences in these variables in the experimental group. Moreover, the 2-by-2 mixed-model analysis of variance indicates a statistically significant group-by-time interaction for all development quotients (global and partial) (*p* < 0.001).

**Figure 2:**
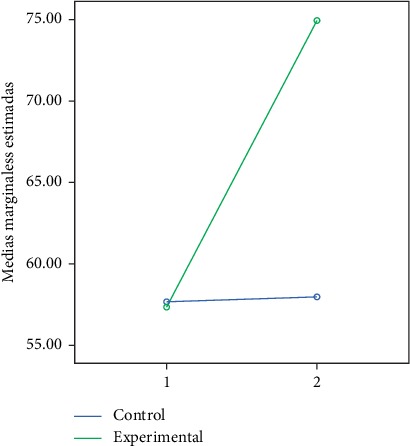
Quotient of global development.

**Figure 3:**
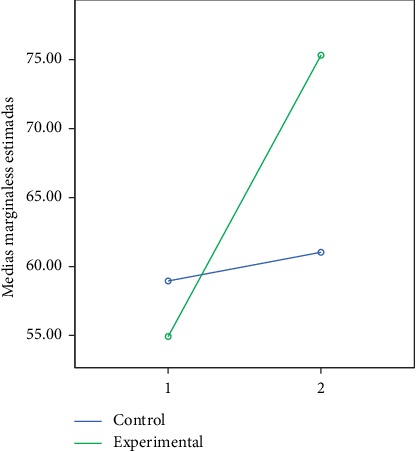
Quotient of motor development.

**Figure 4:**
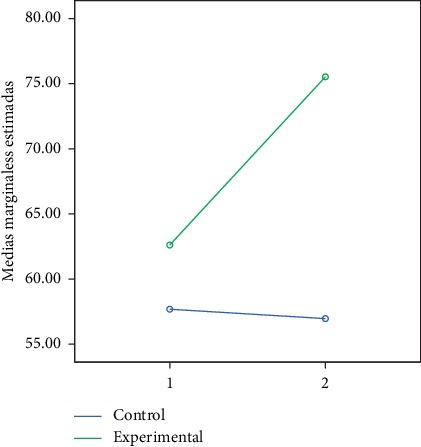
Quotient of visual-motor coordination development.

**Figure 5:**
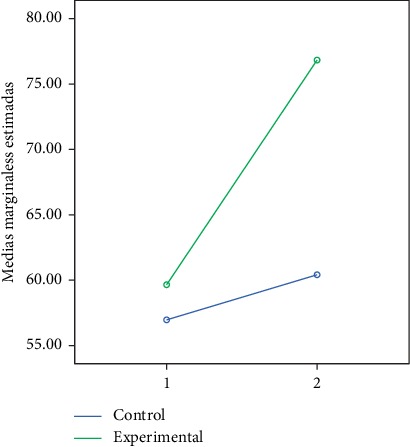
Quotient of language development.

**Figure 6:**
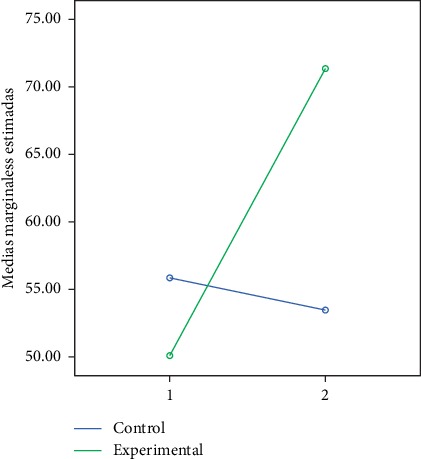
Quotient of social development.

In the following figures, we can observe the marginal averages of the control and experimental group in relation to the global and partial development quotient (Figures 2–6).

## 4. Discussion

Regarding the results between pretest and posttest for partial and global developmental age, the 5-week therapy led to significant changes in global developmental age, motor developmental age, visual-motor coordination age, and language developmental age. There were no significant differences in social developmental age from the control group. However, there were significant differences in the partial and global developmental age in the experimental group. These results indicate that both groups had improvements in the study period regardless of having received infant massage therapy or not. The control group also had improvements for evolutionary reasons because children mature as time progresses. Moreover, both groups continued to attend early care sessions to improve different aspects of child development.

In contrast, there were significant differences in the quotients (global development, motor development, visual-motor coordination, language development, and social development) in the experimental group but not in the control group. This difference in the developmental age (partial and global) and development quotient (partial and global) is because each development quotient of the Brunet-Lézine Early Childhood Psychomotor Development revised scale is calculated by dividing the age of development by the chronological age of the child, multiplied by 100. It is known that babies from the control group had improvements in developmental age, but this improvement did not accompany the chronological age, and thus the development quotient was increased significantly. In contrast, in the experimental group, developmental age was increased to the same extent of chronological age and, for this reason, the development quotients were also increased, probably because of the infant massage performed in this group.

Infant massage produced significant differences in the experimental group compared to the control group. There were significant differences in all variables in the experimental group, considering age (global development, motor development, visual-motor coordination, language development, and social development) and quotients (global development, motor development, visual-motor coordination, language development, and social development). This result indicates that developmental age was significantly higher in the experimental group compared with the control group. The development quotients did not increase in the control group, and the social development quotient decreased in this group. The cause for the decrease in the social development quotient in the control group is unknown. However, our results indicate that infant massage is effective in increasing the developmental age and development quotient of babies with Down syndrome. It seems that the contact and caresses, the look, the communication, and the dedication time of the parents improve the development of babies with Down syndrome. All the elements of the emotional bond are present during infant massage, which can be triggering mechanisms that promote change in the development of the baby [13].

To the best of our knowledge, few studies to date have investigated infant massage therapy in babies with Down syndrome, thus limiting the possibility of comparison between studies.

Hernández-Reif et al. [10] evaluated the effectiveness of infant massage in 21 children with Down syndrome with a mean age of 24.5 ± 9.5 months, divided into an intervention and control group. The experimental group received infant massage for 0.5 hours twice a week, while the control group received reading sessions during the same period. The authors observed changes in all evaluated children because all children attended an early care program. However, the experimental group showed better fine and gross motor skills than the control group. These authors proposed that future studies should ensure that the control group does not receive any type of intervention and that this group should be assigned to a waiting list. In this study, changes were observed in both groups because all babies received early childhood intervention, as reported by Hernández-Reif et al. [10]. At present, not providing early childhood intervention to the control group is considered unethical because it is known that these programs contribute to the development of children and therefore they do not usually remain on the waiting list for a long time. In the present study, the early care intervention for babies with Down syndrome was controlled by the constancy technique because the babies of both groups attended this program.

Campos Serrano et al. [16] found that infant massage did not significantly affect the psychomotor development of healthy babies. Although our sample consisted of babies with Down syndrome, infant massage may affect psychomotor development because the experimental group had significant changes in partial and global developmental age and partial and global development quotients compared with the control group. In this respect, Purpura et al. [1] observed that infant massage affected the development of babies with Down syndrome by accelerating development, especially of visual functions. Environmental enrichment, in the tested form of infant massage, seems to affect maturation of visual functions in all babies, including infants with developmental disorders, because it is applied during a period of high brain plasticity.

Lima [11] evaluated the benefits of Shantala massage in babies with Down syndrome. The sample consisted of five mother-baby pairs. The author observed that the massage favored the onset of certain movements in the infant sooner than expected, indicating that their developmental age was improved, and these results agree with ours. The technique was applied with the same frequency as in our study and with very similar movements, although in a smaller sample. Similarly, Silva et al. [12] reported that Qigong massage improved motor development scores in children with Down syndrome and was an alternative intervention for reducing the impact of disability.

A strength of this work, unlike other studies of infant massage and Down syndrome, is that we have analyzed all areas of development in very young babies with Down syndrome. This has considerable clinical relevance for education and health programs for families with babies with Down syndrome. Infant massage is postulated as a tool to improve child development in the first months of life, as a complement to early childhood care programs.

### 4.1. Limitations and Prospective

One limitation of this study is not having compared the results based on maternal education levels, as well as the sociodemographic situation in families with Down syndrome. Another limitation is not knowing the effect of long-term infant massage in this population.

On the other hand, it is proposed to expand the studies of infant massage by analyzing new variables that may also influence the development and maturation of children, for example, through neuroimaging tests.

## Figures and Tables

**Table 1:** Pretest, demographic, and clinical characteristics of babies.

Variable	Control group *n* = 16	Experimental group *n* = 16	*p* value
Chronological age (days)	136.00 (124.50–206.75)	141.00 (124.00–162.75)	0.836^a^

Gender, *n* (%)			
Boys	11 (68.7%)	10 (62.59%)	0.710^b^
Girls	5 (31.3%)	6 (37.5%)	

Siblings, *n* (%)			
No	8 (50.0%)	7 (43.7%)	0.723^b^
Yes	8 (50.0%)	9 (56.3%)	

Interviewed parents, *n* (%)			
Father	2 (12.5%)	2 (12.5%)	0.700^c^
Mother	14 (87.5%)	14 (87.5%)	

Age of pretest global development, median (IQR)	75.00 (57.75–138.00)	82.50 (66.75–106.50)	0.669^a^

Age of pretest motor development, median (IQR)	75.00 (60.00–146.25)	75.00 (62.50–107.50)	0.985^a^

Age of pretest visual-motor coordination development, median (IQR)	85.00 (50.00–137.50)	89.00 (80.00–111.00)	0.564^a^

Age of pretest language development, median (IQR)	60.00 (60.00–146.25)	97.50 (60.00–116.25)	0.669^a^

Age of pretest social development, median (IQR)	70.00 (48.75–137.50)	70.00 (60.00–90.00)	0.926^a^

Quotient of pretest global development, mean (SD)	57.67 (16.82)	57.35 (11.42)	0.949^d^

Quotient of pretest motor development, mean (SD)	58.95 (16.66)	54.92 (12.32)	0.443^d^

Quotient of pretest visual-motor coordination development, mean (SD)	57.68 (18.57)	62.61 (11.47)	0.375^e^

Quotient of pretest language development, mean (SD)	56.96 (17.19)	59.65 (15.32)	0.664^d^

Quotient of pretest social development, mean (SD)	55.86 (18.97)	50.10 (12.42)	0.318^d^

^a^Mann–Whitney *U* test was used. ^b^Pearson's chi-square test was used. ^c^Fisher's exact test was used. ^d^Student's *t*-test was used for independent samples. ^e^Welch's *t*-test was used. IQR: interquartile range.

**Table 2:** Pretest-posttest contrast within each group and between groups for partial and global developmental age.

Variable	Group	Pretest median (IQR)	Posttest median (IQR)	Within-groups comparison *p* value	Within-groups change scores median (IQR)	Between-groups change scores *p* value
Age of global development	Control	75.00 (57.75–138.00)	102.00 (74.25–174.75)	**<0.001** ^**a**^	13.50 (9.00–29.25)	**<0.001** ^**a**^
	Exp	93.38 (36.33)^b^	146.62 (35.66)^b^	**<0.001** ^**c**^	49.50 (39.75–68.25)	

Age of motor development	Control	75.00 (60.00–146.25)	110.00 (75.00–182.75)	**<0.001** ^**a**^	20.00 (14.75–37.50)	**<0.001** ^**a**^
	Exp	75.00 (62.50–107.50)	135.00 (110.00–188.00)	**<0.001** ^**a**^	52.50 (41.00–65.00)	

Age of visual-motor coordination development	Control	85.00 (50.00–137.50)	115.00 (65.00–170.00)	**0.001** ^**a**^	10.00 (10.00–30.00)	**<0.001** ^**a**^
	Exp	100.56 (34.07)^b^	147.75 (36.63)^b^	**<0.001** ^**c**^	41.50 (29.50–67.25)	

Age of language development	Control	60.00 (60.00–146.25)	105.00 (90.00–172.50)	**0.001** ^**a**^	26.25 (16.88)	**<0.001** ^**c**^ **mean difference** **=** **27.19 CI (13.64; 40.74)**
	Exp	96.56 (37.14)^b^	150.00 (39.49)^b^	**<0.001** ^**c**^	53.44 (20.47)	

Age of social development	Control	70.00 (48.75–137.50)	80.00 (70.00–147.50)	0.068^**a**^	14.69 (29.52)^b^	**<0.001** ^**c**^ **mean difference** **=** **41.87 CI (24.11; 59.64)**
	Exp	70.00 (60.00–90.00)	130.00 (122.50–147.50)	**<0.001** ^**a**^	56.56 (18.41)^b^	

^a^Mann-Whitney *U* test was used. ^b^Mean and standard deviation (SD) are shown. ^c^Student's *t*-test was used for related samples. ^d^The difference between groups and their confidence interval (CI) are shown. IQR: interquartile range. Exp: experimental group.

**Table 3:** Pretest-posttest contrast within each group and between groups for partial and global developmental quotient.

Variable	Group	Pretest mean (SD)	Posttest mean (SD)	Within-groups change scores		Between-groups change scores	2-by-2 mixed model		
				Mean difference CI	*p* value	Mean difference CI	Time pretest vs posttest *F* *p* value *η*^2^	Main effect of group *F* *p* value *η*^2^	Interaction effect *F* *p* value *η*^2^
Quotient of global development	Control	57.67 (16.82)	57.97 (15.66)	0.30 (-4.45; 5.05)	0.894^a^	17.29 (9.19; 25.39)	*F* **=** **20.35** **< 0.001** *η*^2^ **=** **0.40**	*F* **=** **767.84 < 0.001** *η ***^2^** **=** **0.96**	*F* **=** **18.9< 0.001** *η ***^2^** **=** **0.39**
	Exp	57.35 (11.42)	74.94 (10.38)	17.59 (10.59; 24.59)	**< 0.001** ^**a**^				

Quotient of motor development	Control	58.95 (16.66)	61.03 (15.17)	2.08 (-2.24; 6.39)	0.321^a^	18.32 (9.71; 26.92)	*F* **=** **28.42** **< 0.001** *η ***^2^** **=** **0.49**	*F* **=** **780.61** **< 0.001** *η ***^2^** **=** **0.96**	*F* **=** **18.88 < 0.001 ***η ***^2^** **=** **0.39**
	Exp	54.92 (12.32)	75.32 (11.15)	20.40 (12.51; 28.27)	**< 0.001** ^**a**^				

Quotient of visual-motor coordination development	Control	57.68 (18.57)	56.96 (16.68)	-0.72 (-7.14; 5.69)	0.814^a^	13.64 (3.99; 23.28)	*F* **=** **6.68** **0.015** *η ***^2^** **=** **0.18**	*F* **=** **734.14** **< 0.001** *η ***^2^** **=** **0.96**	*F* **=** **8.35** **0.007** *η ***^2^** **=** **0.22**
	Exp	62.61 (11.47)	75.53 (10.94)	12.92 (5.17; 20.67)	**0.003** ^**a**^				

Quotient of language development	Control	56.97 (17.19)	60.41 (16.51)	3.44 (-1.84; 8.73)	0.185^a^	13.74 (4.79; 22.67)	*F* **=** **22.22** **< 0.001** *η ***^2^** **=** **0.43**	*F* **=** **607.81** **< 0.001** *η ***^2^** **=** **0.95**	*F* **=** **9.85** **0.004** *η ***^2^** **=** **0.25**
	Exp	59.65 (15.32)	76.83 (14.10)	17.18 (9.49; 24.87)	**< 0.001** ^**a**^				

Quotient of social development	Control	55.86 (18.97)	53.47 (19.28)	-2.39 (-10.09; 5.31)	0.519^a^	23.65 (13.45; 33.84)	*F* **=** **14.29 0.001 ***η ***^2^** **=** **0.32**	*F* **=** **494.46** **< 0.001** *η ***^2^** **=** **0.94**	*F* **=** **22.43** **< 0.001** *η ***^2^** **=** **0.43**
	Exp	50.10 (12.42)	71.36 (13.24)	21.26 (13.92; 28.59)	**< 0.001** ^**a**^				

^a^Student's *t*-test was used for related samples. SD: standard deviation. CI: confidence interval. Exp: experimental group.

## Data Availability

The database used to support the findings of this study is restricted by the Data Protection Law in order to protect Patient Privacy. Data are available from E. Pinero-Pinto (epinero@us.es) or J.- J. Jiménez-Rejano (jjimenez@us.es) for researchers who meet the criteria for access to confidential data.

## Supplementary Materials

Supplementary Materials The CONSORT Checklist comprises 25 items. This checklist items focus on reporting how the trial was designed, analyzed, and interpreted.
